# Role of time in binding features in visual working memory

**DOI:** 10.1037/rev0000331

**Published:** 2022-01-31

**Authors:** Sebastian Schneegans, Jessica M. V. McMaster, Paul M. Bays

**Affiliations:** Department of Psychology University of Cambridge Cambridge UK

**Keywords:** visual working memory, feature binding, dual report task, swap errors

## Abstract

Previous research on feature binding in visual working memory has supported a privileged role for location in binding an object’s non-spatial features. However, humans are able to correctly recall feature conjunctions of objects that occupy the same location at different times. In a series of behavioral experiments, we investigated binding errors under these conditions, and specifically tested whether ordinal position can take the role of location in mediating feature binding. We performed two dual-report experiments in which participants had to memorize three colored shapes presented sequentially at the screen center. When participants were cued with the ordinal position of one item and had to report its shape and color, report errors for the two features were largely uncorrelated. In contrast, when participants were cued e.g. with an item’s shape and reported an incorrect ordinal position, they had a high chance of making a corresponding error in the color report. This pattern of error correlations closely matched the predictions of a model in which color and shape are bound to each other only indirectly via an item’s ordinal position. In a third experiment, we directly compared the roles of location and sequential position in feature binding. Participants viewed a sequence of colored disks displayed at different locations, and were cued either by a disk’s location or its ordinal position to report its remaining properties. The pattern of errors supported a mixed strategy with individual variation, suggesting that binding via either time or space could be used for this task.

## Role of time in binding features in visual working memory

Our visual world is composed of objects that are characterized by a combination of visual features. To faithfully memorize a scene, we need to encode not only the individual features that are present, but also their specific combinations that constitute different objects. Both in our everyday experience and in psychophysical experiments, objects are often separated in space, and it has long been recognized in the psychological literature that location plays a special role in individuating objects and mediating the binding between their features in working memory (see Schneegans & Bays, 2019 for review). But in a dynamic world, we can also perceive different objects at the same location, separated in time. The mechanism of feature binding in visual working memory for sequentially presented objects, and the role that presentation time plays in mediating binding in working memory, have received less attention. In the present study, we adapted methods previously used to investigate the role of space in feature binding to elucidate the role of time, and the interaction between space and time in binding.

Strong evidence for a special role of space for feature binding in working memory came from the study of Treisman and Zhang (2006), who observed that task-irrelevant location changes led to decreased performance and specific response biases in change detection tasks. Several more recent studies employed delayed reproduction tasks in a dual-report paradigm to investigate feature binding and the nature of object representations in working memory. In this type of task, participants view an array of sample stimuli characterized by multiple features (e.g., colored oriented bars). After a brief delay, a cue is presented that identifies the target item from the sample array, and participants have to report two features of that item on a continuous scale (e.g. by adjusting a color, orientation, or location probe). This form of response makes it possible to distinguish between different types of errors, and to detect dependencies between errors in the two responses. Of particular interest for investigating feature binding are *swap errors*, in which participants report the feature of an item from the sample array other than the cued target item.

**Figure 1:**
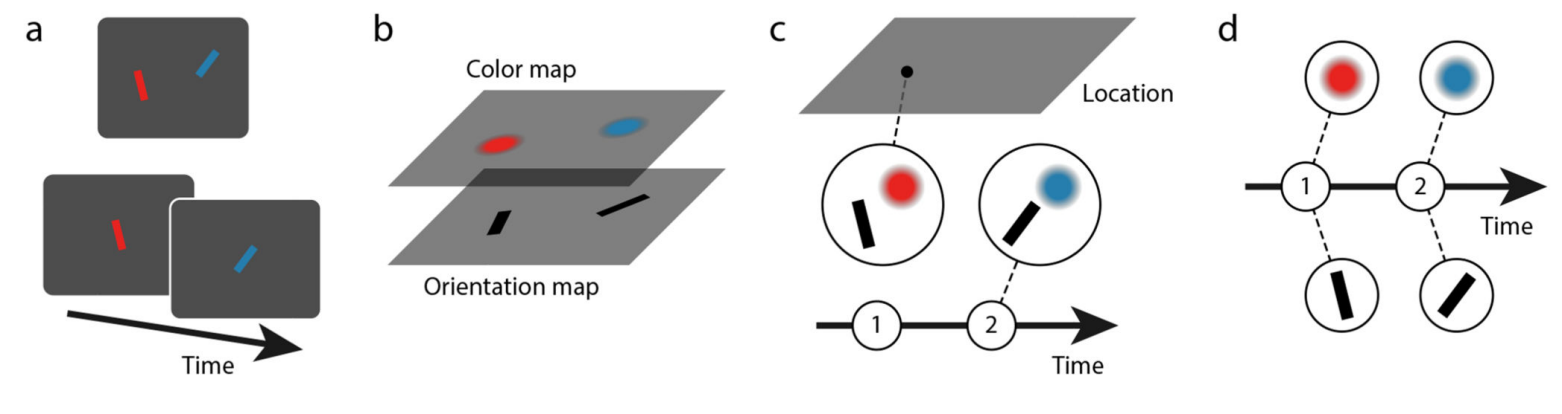
Conceptual models of feature binding. (a) Example stimulus displays with color-orientation conjunctions, either presented simultaneously at different locations (top) or sequentially at the same location (bottom). (b) Spatial binding model with separate feature maps over visual space. Different features of an object are bound to each other only indirectly via their shared location. Each item’s ordinal position or presentation time may also be encoded in an additional feature map. (c) Object-based model. The visual features of an object are bound directly to each other, and the whole object can be bound to a location (as shown for the red object) and/or a point in time (blue object). (d) Temporal binding model. Object features, and potentially also object locations, are bound independently to a point in time or an ordinal position.

Multiple studies using this paradigm have consistently found that when the target item is cued by its location in the sample array, errors in reporting its color and orientation occur independently (Fougnie & Alvarez, 2011; Fougnie, Cormiea, & Alvarez, 2013), and this is true for swap errors in particular (Bays, Wu, & Husain, 2011). In contrast, when the target item is cued by another feature like color or orientation, correlated swap errors are observed. More specifically, when a swap error occurs in reporting the location of the cued item, participants also show a strong tendency to report the other features of the non-target item at the reported location (Kovacs & Harris, 2019; Schneegans & Bays, 2017). This supports the idea that non-spatial features of an object are bound to each other only indirectly via their shared location. In the neural system, this may be realized through separate feature maps over visual space (Schneegans & Bays, 2017; Schneegans, Spencer, & Schöner, 2016; Treisman, 1988). We will refer to this as the spatial binding model (Figure 1b).

This account of feature binding contrasts with conceptualizations of working memory that are based on bound object representations. For instance, the influential object file theory (Kahneman, Treisman, & Gibbs, 1992; Treisman & Zhang, 2006) considers feature maps to be the basis of sensory representations, and assumes that location takes an important role in forming bound representations through attentional selection of single objects. But once the features of an item are combined into an object file, location is no longer required for maintaining the binding of other visual features (illustrated in Figure 1c). Similarly, slot models of working memory (Luck & Vogel, 1997) assume that bound object representations comprising all features of a visual stimulus are the natural units of working memory, without any special role for location.

An important limitation of the spatial binding account is that it cannot readily explain how we can memorize the feature combinations of multiple objects that are presented sequentially at the same location. While there is evidence that memory performance is impaired when stimuli are presented sequentially (Allen, Baddeley, & Hitch, 2006; Gorgoraptis, Catalao, Bays, & Husain, 2011), multiple items shown at the same location can still be memorized separately, and ordinal position can be used as an effective cue to select one item (e.g. Harrison & Tong, 2009). One previous study reported that feature binding is selectively impaired when sample stimuli are presented sequentially at the same location rather than at different locations, even if location is not task-relevant (Pertzov & Husain, 2014). However, a recent replication study found that this effect did not generalize when longer inter-stimulus intervals or different feature combinations were used, and attributed it to perceptual interference (Schneegans, Harrison, & Bays, 2021). Thus, a shared location of multiple sample items does not appear to create specific disruptions of binding memory.

A possible explanation that reconciles this finding with a spatial binding account is that stimuli presented sequentially at the same location are internally remapped to different locations, such that binding via space becomes feasible again (Abrahamse, Van Dijck, Majerus, & Fias, 2014; van Dijck, Abrahamse, Majerus, & Fias, 2013). In this account, an ordinal position may also be associated with each location, possibly in the form of another feature map over visual space, to allow an item’s ordinal position to be recalled or used as retrieval cue for other features.

An alternative explanation is that the time at which different stimuli are perceived, or their temporal order, can take a similar role as space in binding visual features. Some parallels between the roles time and space have been observed for feature binding in visual perception. When participants briefly view an array of colored letters with a simultaneously presented location cue, report errors for the color and identity of the cued item are largely independent (which matches the observation in working memory described above); the same independent report errors are found when participants view a rapid stream of colored letters at a single location, and a temporal cue in the form of briefly flashed ring is used (Vul & Rich, 2010). In visual working memory, it has been observed that participants can encode color-shape conjunction when the features are presented either spatially or temporally separated, with only modest reduction in performance compared to unified sample stimuli, and no reliance on central attentional resources (Karlsen, Allen, Baddeley, & Hitch, 2010).

Memory for the temporal order of stimuli has been studied in great detail in the domain of verbal working memory (Marshuetz, 2005). Patterns of recall errors identified in this field, such as gradients in the frequency of transpositions (the equivalent of swap errors) with temporal distance, have also been shown to generalize to sequential recall in visuo-spatial memory (Guérard & Tremblay, 2008; see Hurlstone, Hitch, & Baddeley, 2014, for a comprehensive review of this field). Performance is similar in recall of sequences of locations (Corsi block tapping task) and matched sequences of simple verbal memory items, such as digits (Monaco, Costa, Caltagirone, & Carlesimo, 2013). However, sequential order does not appear to take the same central role in retrieval from visuo-spatial memory as it does for verbal memory (Gmeindl, Walsh, & Courtney, 2011). Nonetheless, recall performance in reporting the sequential order of visual stimuli is similar to performance for reporting the objects’ locations (Delogu, Postma, & Nijboer, 2012), and both sequential order and location are encoded in working memory automatically even when not task relevant (Heuer & Rolfs, 2021).

An explicit role of temporal order in binding features in visual working memory has first been proposed in the context of rapid serial visual presentation tasks, namely to explain specific misbinding errors related to the attentional blink effect. Wyble, Bowman and colleagues proposed a model in which representations of feature conjunctions are formed by linking multiple individual features to one out of a limited set of *tokens*, which explicitly encode the temporal order of stimuli (Bowman & Wyble, 2007; Wyble, Bowman, & Nieuwenstein, 2009; Wyble, Potter, Bowman, & Nieuwenstein, 2011).

The binding pool model (Swan & Wyble, 2014) builds on this ideas to explain performance in a variety of visual working memory tasks, where it assumes that items are attended and encoded one at a time even when stimulus arrays are presented simultaneously. All of the item’s visual features (including its location) are encoded in the activity of a pool of neurons with mixed selectivity and associated to a token reflecting an ordinal position, and this token mediates the coherent re-activation of the associated features at retrieval. We can conceptualize this type of account as *temporal binding*, in which the binding between visual features is mediated by the time at which a stimulus was encoded (Figure 1d; note that the binding pool model is not a pure example of this, as it also employs direct conjunctive coding of feature combinations).

To adjudicate between these accounts and shed light on the mechanisms of feature binding for sequentially presented objects, we adapted the kind of dual-report task previously used for simultaneously presented sample arrays. The experiment design and analysis follows the same rationale employed in Schneegans and Bays (2017): If the two stimulus features that are to be reported are bound separately and directly to the cue feature, then report errors should occur independently. In contrast, if one of the reported features is bound to the cue only indirectly via the other one, then correlated errors should occur, especially in the case of swap errors.

In Experiment 1, we compare temporal binding and object-based accounts in two working memory tasks in which sample items are presented sequentially at the same location. In Experiment 2, we present items sequentially and at different locations, to determine whether either presentation time or location are dominant in mediating binding between visual features, or if the neural system can switch between different binding mechanisms.

In all experiments, we present stimuli at fixed and equal intervals, and operationalize time as the ordinal position at which each item appears in the sequence. We take this approach to avoid making any strong assumptions about how continuous time is represented in the neural system, a question which we consider to be outside of the scope of the present study (see Manohar, Pertzov, & Husain, 2017 for review).

## Experiment 1

**Figure 2:**
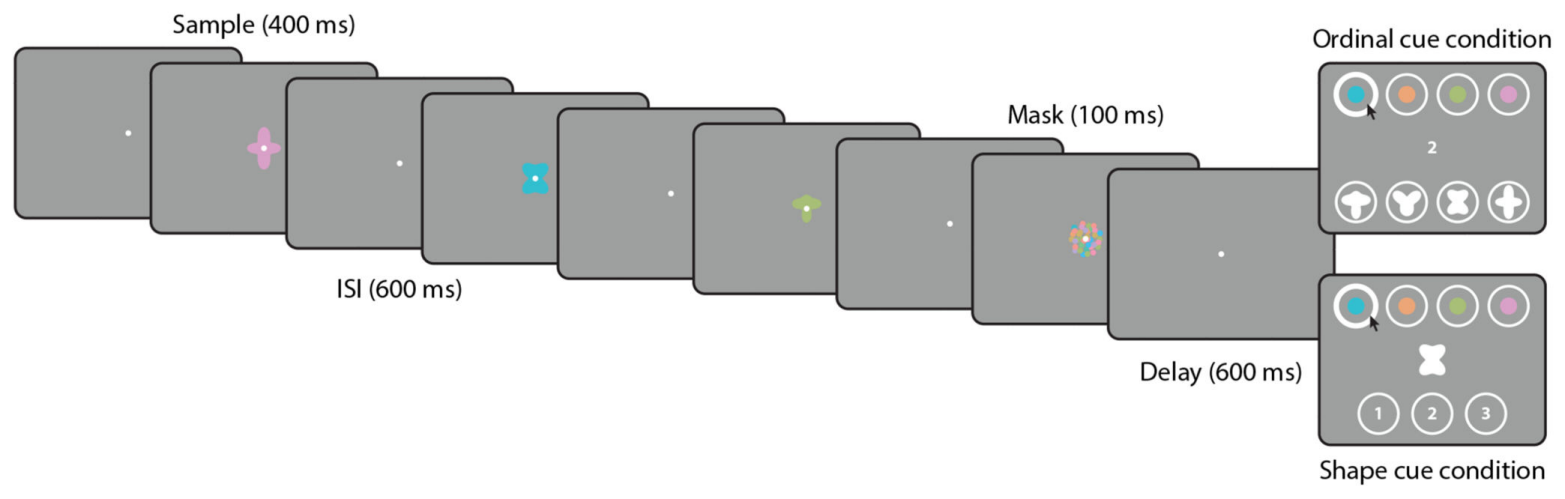
Behavioral task in Experiment 1a. The presentation of the sample stimuli is identical in the two task conditions, only the response cue and response options differ.

In two dual-report tasks, participants viewed three sequentially presented colored shapes (Figure 2). They were then either cued with the ordinal position of one sample item and had to report its features (color and shape); or they were cued with one of these features and had to report both the remaining feature and the ordinal position of the cued item. We investigated the mechanisms of feature binding in these tasks by analyzing error correlations between the two responses.

We employed continuous feature spaces for color and shape, both to retain a close link to previous dual-report experiments investigating feature binding in visual working memory (Bays et al., 2011; Fougnie & Alvarez, 2011; Fougnie et al., 2013; Schneegans & Bays, 2017), and to discourage verbal encoding. In Experiment 1b, we further added a concurrent articulatory suppression task to rule out contributions from verbal memory to the performance in the main task. The present work deviates from most previous studies in that responses were made by selecting from a set of discrete choices, rather than adjustment on a continuous scale (but see Fougnie & Alvarez, 2011). This was done to make color and shape responses more comparable to the ordinal position response, which is necessarily discrete, and to avoid any incentive for participants to treat ordinal position differently from color or shape merely due to the response mode.

### Methods

#### Participants

Ten participants (3 male, 7 female, age 23.5 ± 2.6 years [M ± SD]) performed Experiment 1a after giving informed consent in accordance with the declaration of Helsinki, and a separate group of ten participants (2 male, 8 female, age 25 ± 3.7 years) performed Experiment 1b. All participants reported normal or corrected-to-normal visual acuity, and demonstrated normal color vision in an Ishihara color test. The experiments were completed in a single session lasting one to one and a half hours, and participants received monetary compensation of 10 GBP per hour. Procedures were approved by the University of Cambridge Psychology Research Ethics Committee.

#### Stimuli

The memory sample stimuli in Experiment 1 were colored shapes, with both colors and shapes drawn from continuous circular feature spaces. Colors were drawn from a color wheel in CIELAB color space with a fixed luminance of 74, centered at [0, 0] in the a-b plane, and with a radius of 40. Individual hue values are specified by an angle on this color wheel.

Shapes were generated as weighted combinations of radial sinusoids. At each angle *θ*, the distance of the shape’s outline to the midpoint of the shape was determined as

(1) $$r(\theta )=r_{0}+\frac{r_{0}}{2}\sum_{i}w_{i}\cos\left(f_{i}\theta +\phi_{i}\right),$$

where *r*_0_ is the base radius, *w_i_* is the weight of each sinusoid, *f_i_* its frequency and *ϕ_i_* its phase offset. We used four sinusoids with frequencies (2, 3, 4, 4) and phase offsets (0, 0, 0, π). Individual shapes were generated by varying the weights *w_i_*. For a given shape angle *α* in the circular feature space of possible shapes, the weights were determined as

(2) $$w_{i}\text{=}\{\begin{array}{ll} \frac{\cos\left(2\alpha -c_{i}\right)+1}{2}, & \text{ if }D_{o}\left(\alpha -\frac{c_{i}}{2}\right)\leq \frac{\pi }{2}\\ \text{0}, & \text{ otherwise, } \end{array}$$

with *c_i_* = π(i − 1). Here, *D_o_* denotes angular distance on a circle, yielding a value in the range [0, *π*]. The size of the shapes was controlled by the base radius *r*_0_, which we set to 1.25 degrees of visual angle (dva).

The color angles and shape angles of different items within each trial (including the foil feature presented as a response option, see below) differed by at least 60°.

#### Procedure

Participants were seated in front of the display (27” LCD screen, resolution 2560 × 1440, 144 Hz) at a viewing distance of 60cm, with their head stabilized by a head rest. Gaze direction was continuously monitored by an infrared eye tracker (Eyelink 1000, operating at 1000Hz). Stimulus presentation, response collection and eye tracking were controlled using Matlab (The MathWorks, Inc.) with the Psychophysics Toolbox (Brainard, 1997; Kleiner, Brainard, & Pelli, 2007; Pelli, 1997) and Eyelink Toolbox (Cornelissen, Peters, & Palmer, 2002) extensions.

Participants initiated each trial by clicking the mouse button. A white fixation point (diameter 0.25 dva) was shown at the center of the screen, and once stable fixation was detected (gaze within 2 dva of the fixation point for 500 ms), the memory sample stimuli were presented.

In Experiment 1a, participants viewed three colored shapes, each presented at the screen center for 400 ms, and followed by a blank screen for 600 ms. The final blank interval was followed by a mask stimulus composed of small colored disks, shown for 100 ms (diameter 3.75 dva, with colors drawn randomly from the color wheel). After another 600 ms delay, a cue was presented at the screen center. In the *ordinal cue condition*, the cue was one of the digits 1, 2 or 3, indicating which item in the sequence had to be reported. In the *shape cue condition*, the cue was a white shape matching one of the three shapes shown as sample stimuli.

Participants initiated their response by moving the mouse, upon which the response options were shown within white circles above and below the fixation point (no earlier than 500 ms after cue onset to reduce interference from the response display; Souza, Rerko, & Oberauer, 2016). In both conditions, four colored disks appeared horizontally aligned above the fixation point. These included the three colors of the sample stimuli and one foil color, arranged in randomized order. In the ordinal cue condition, four white shapes appeared below the fixation point, likewise including the three sample stimulus shapes and one foil in randomized order. In the shape cue condition, the three digits 1, 2 and 3 were displayed instead. Participants reported the features and/or ordinal position of the cued item by clicking first on one of the color response fields, then on one of the other response fields (shape or ordinal position). The response order was enforced by the experiment software, in that clicking on one of the bottom response fields before a color was selected would have no effect.

In Experiment 1b, the memory sample stimuli remained the same, but the roles of color and shape in the response phase were swapped. In the *ordinal cue* condition, the cue was a digit as before, and in the *color cue* condition, the cue was a colored disk presented at the screen center. The first report in both cases was the shape of the cued item, to be selected out of four options displayed above the fixation point. The second report in the ordinal cue condition was the cued item’s color, and in the shape cue condition it was the cued item’s ordinal position, with response options displayed below the fixation point.

In addition, Experiment 1b introduced a concurrent articulatory suppression task. Before the presentation of the sample stimuli, three letters were presented at the screen center for 1000 ms, randomly chosen for each trial, followed by a 1000 ms blank interval. The participants had to repeat this sequence of letters aloud throughout the sample and delay periods until the cue was shown, at a rate of one to two letters per second (monitored online by the experimenter). To avoid interference from the articulation on eye tracking, the chin rest used in Experiment 1a was removed and head position was stabilized only by a forehead rest.

In both experiments, participants had to maintain fixation on the central fixation point throughout the sample and delay period, to ensure that all samples were viewed equally (the fixation point remained visible during the blank intervals and on top of the sample stimuli). If fixation was lost before the presentation of the cue (beyond brief blinks of no more than 200 ms), the current trial was aborted and a new trial started. Conditions were blocked in both experiments, and participants completed three consecutive blocks of 36 trials for each task condition (108 trials per condition in total). Within each block, the first, second and third item in the sequence were cued as the target equally often. The order of conditions was balanced across participants.

#### Analysis

The discrete responses in this task can be classified into target responses (choosing the correct feature), swap responses (choosing a feature that was present in the trial, but does not belong to the cued target item), and foil responses (choosing the feature that was not present in the trial). When we consider the two responses made in each trial together, we can further distinguish between congruent swaps (the features or ordinal position of the same non-target item are chosen) and incongruent swaps. We used the proportion of target responses as a measure of overall recall performance, dependent on the reported feature and the ordinal position of the cued item (since we can expect to observe higher performance for more recently viewed sample items). We compared the proportions of swap and foil responses to determine whether responses in error trials were simply random, or whether they reflected specific failures to retrieve the feature binding correctly while at least partial memory of the individual features present in a trial was maintained.

To elucidate the mechanism of feature binding in this task, we fit the behavioral data with two models that make specific and opposing predictions about error correlations between the two responses in each trial. Both models are defined through their confusion matrices, which specify the probabilities that a participant will report, for instance, the color of the first item and the shape of the second item when cued to report the features of the second item in the sequence. This yields a three-dimensional matrix **P** for each task condition, with entries

(3) $$p(i,j,k)=\mathrm{Pr}\left(m_{\text{R}1}=i,m_{\text{R}2}=j|m_{\text{C}}=k\right),$$

where Pr(*x*|*y*) indicates the conditional probability of *x* given *y*. We denote with *m*_R1_, *m*_R2_ and *m*_C_ the ordinal indices of the selected option for the first and second report feature, and the given cue feature. These can take values 1 to 3 (for features that were present in the trial’s sample stimuli) or 4 (for the foil feature, in shape and color responses only).

The *temporal binding model* assumes that memory representations for color and shape of an item are bound to each other only indirectly via the item’s ordinal position. In the ordinal cue condition, the choice of response options should then occur independently of each other, such that

(4) $$\mathrm{Pr}\left(m_{\text{col }},m_{\text{shp }}|m_{\text{ord }}\right)=\mathrm{Pr}\left(m_{\text{col }}|m_{\text{ord }}\right)\mathrm{Pr}\left(m_{\text{shp }}|m_{\text{ord }}\right).$$

In the shape cue condition, the cue is used first to retrieve the ordinal position of the cued item (even though this is only reported in the second response), and then the retrieved ordinal position is used as a secondary cue to retrieve the item’s color, yielding

(5) $$\mathrm{Pr}\left(m_{\text{col }},m_{\text{ord }}|m_{\text{shp }}\right)=\mathrm{Pr}\left(m_{\text{col }}|m_{\text{ord }}\right)\mathrm{Pr}\left(m_{\text{ord }}|m_{\text{shp }}\right).$$

The *object-based model* assumes that the features (color and shape) are bound directly to each other, and the object formed by the conjunction of its features is then bound to an ordinal position. There are different ways how this conceptual model could be realized. For ease of comparison, we chose an implementation that is directly symmetrical to the temporal binding model. We assume that the two responses are generated independently in the shape cue condition,

(6) $$\mathrm{Pr}\left(m_{\text{col }},m_{\text{ord }}|m_{\text{shp }}\right)=\mathrm{Pr}\left(m_{\text{col }}|m_{\text{shp }}\right)\mathrm{Pr}\left(m_{\text{ord }}|m_{\text{shp }}\right),$$

since accurate retrieval of the color associated with the cue shape should be unaffected by errors in the ordinal position report. In the ordinal cue condition, the cue is used to select the shape response, and the associated color is then chosen based on the shape,

(7) $$\mathrm{Pr}\left(m_{\text{col}},m_{\text{shp}}|m_{\text{ord}}\right)=\mathrm{Pr}\left(m_{\text{col}}|m_{\text{shp}}\right)\mathrm{Pr}\left(m_{\text{shp}}|m_{\text{ord}}\right).$$

This reflects that swap errors should affect whole bound objects.

In each model, the full three-dimensional confusion matrix for each task condition is determined from a pair of two-dimensional confusion matrices with entries Pr (*m*_F1_ | *m*_F2_). The free parameters of each model are the entries in these confusion matrices for a pair of features. Maximum likelihood fits of these parameters for each participant can be obtained directly by matching the probabilities to the observed response frequencies,

(8) $$\hat{\mathrm{Pr}}\left(m_{\text{F}1}=i|m_{\text{F}2}=j\right)=\frac{|\left\{\text{ trials }t|m_{\text{F}1}(t)=i\wedge m_{\text{F}2}(t)=j\right\}|}{|\left\{\text{ trials }t|m_{\text{F}2}(t)=j\right\}|}.$$

Here, {trials *t* | *c*} is the set of all trials that fulfill condition *c*, and |{. . .}| denotes the cardinality of a set.

In each model, there is one pair of features that appears in the equations for both task conditions (Pr (*m*_col_ | *m*_ord_) for the temporal binding model, Pr (*m*_col_ | *m*_shp_) for the object-based model). For these, we used the pooled frequencies from both task conditions as basis for the estimated confusion matrix. The log-likelihood of each model given a participant’s data can then be determined as sum of the logarithms of entries *p*(*i, j, k*) that correspond to each observed response.

There is one complication in that the shared confusion matrix for Pr (*m*_col_ | *m*_shp_) in the object-based model would need to include the probabilities of reporting colors based on a foil shape, in order to capture trials in the ordinal cue condition in which a foil response occurs in the shape report. These probabilities cannot be shared across task conditions (because a foil shape is never used as cue in the shape cue condition), and no corresponding probabilities exist in the shared matrix Pr (*m*_col_ | *m*_ord_) of the temporal binding model (because there is no foil response option for ordinal position). To allow a fairer comparison between the two models, we excluded all trials with a foil response in the shape report of the ordinal cue condition from the model fits.

### Results of Experiment 1a

#### Recall performance

**Figure 3:**
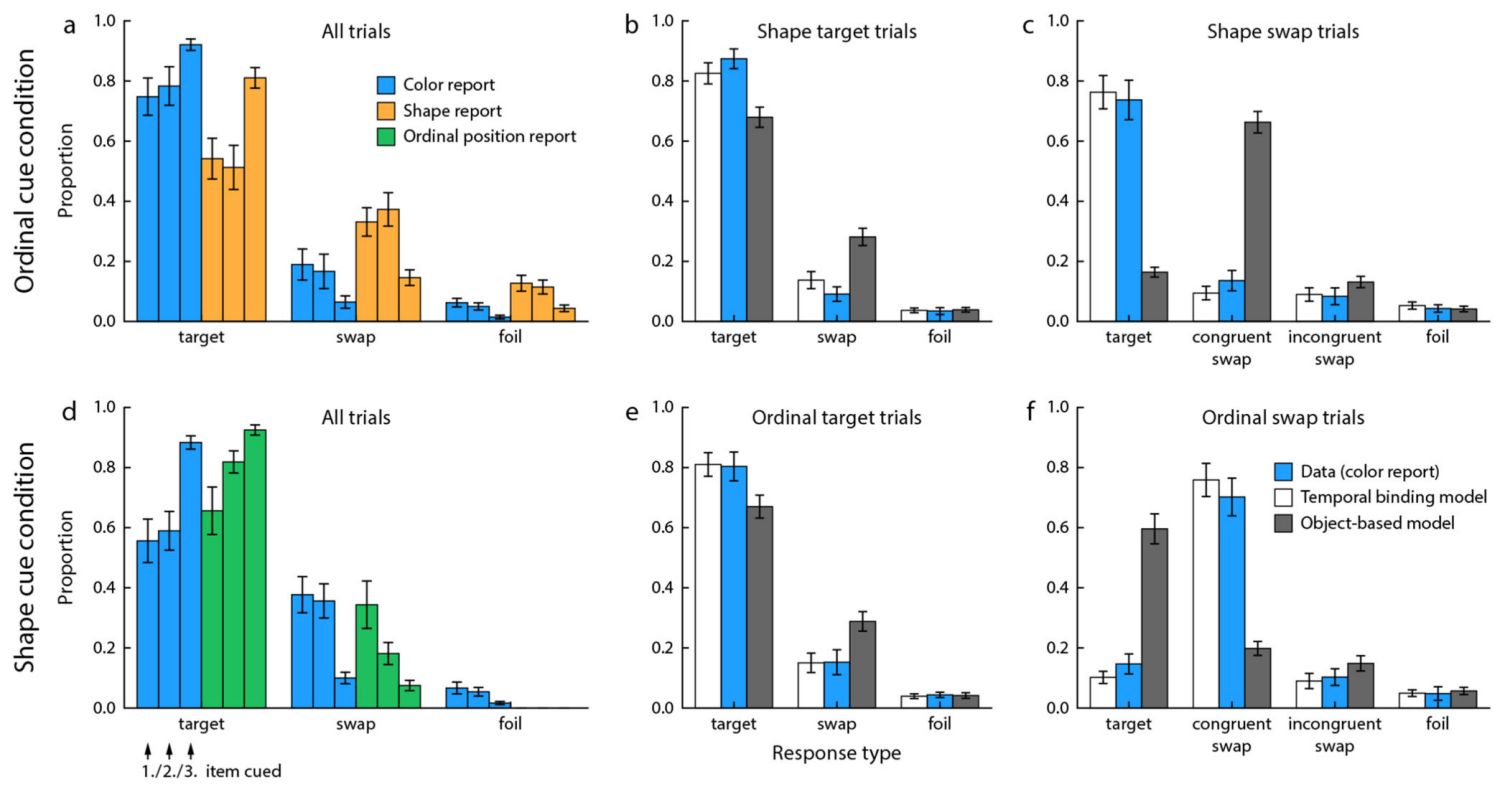
Response distributions in Experiment 1a. (a) Proportions of target, swap and foil responses in the two reports of the ordinal cue condition. The three individual bars for each report and response type show the proportions for each of the three ordinal positions at which the cued target item could appear in the sequence, as indicated in panel d. (b) Proportion of response types for the color report in trials with a correct shape report (collapsed over ordinal positions of the target), and model predictions for these proportions. (c) Proportions of response types for the color report in trials with a swap error in the shape report, and model predictions. Here, we can distinguish between congruent swap errors (features of the same non-target item selected for color and shape) and incongruent swap errors (features of different non-targets selected). (d-f) Corresponding results for the spatial cue condition. Error bars indicate ±1 *SE*.

In Experiment 1a, participants had to memorize three colored shapes presented sequentially at the screen center. We first describe the results for the ordinal cue condition, in which participants had to select the color and shape of a target cued by its position in the sequence. Figure 3a shows proportions of target, swap, and foil responses for each feature, separately for each ordinal position of the target.

We applied a two-way repeated measures ANOVA on the proportion of target responses as a measure of recall performance, with factors report feature (color or shape) and ordinal target position (1 to 3). We found a significant effect of report feature (*F*(1, 54) = 18.2, *p* = 0.002), with higher proportion of target responses for color than for shape. This may be either due to overall better memory for color than shape, or due to the order of responses (color was always reported first). There also was a significant effect of ordinal target position (*F*(2, 54) = 23.0, *p* < 0.001), with better recall performance for more recently viewed items, but no significant interaction between the two factors (*F*(2, 54) = 3.30, *p* = 0.060). For the remaining analyses, we pooled data across ordinal target positions, since the observed recency effect is not the focus of the present study.

We compared the proportions of swap and foil responses for each report feature, to determine whether failure in retrieving the correct bindings significantly contributed to response errors. In the shape report, the proportion of swap responses was significantly higher than expected if errors were completely random, taking into account that there were always two response options counted as swaps and only one foil option (*t*(9) = 4.1, *p* = 0.003). The difference was not significant for the color report (*t*(9) = 1.57, *p* = 0.15).

In the shape cue condition, participants had to report the color and the ordinal position of an item when cued with its shape (Figure 3d). Applying the same analyses, we found that the proportion of target responses was significantly higher for ordinal position than color, despite color being reported first (*F*(1, 54) = 8.04, *p* = 0.020). This was still the case if we excluded the color foil responses for a fairer comparison (given that there is no foil response option in the ordinal position report; *F*(1, 54) = 5.31, *p* = 0.047). There was again a significant effect of target ordinal position (*F*(2, 54) = 20.7, *p* < 0.001) and also a significant interaction (*F*(2, 54) = 5.00, *p* = 0.019), in that the difference in performance for color and ordinal position report was more pronounced when the target was the first or second item in the sequence. In this condition, the proportion of swaps compared to foil responses for color was significantly higher than expected by chance (*t*(9) = 6.15, *p* < 0.001).

Finally, we compared the performance in the color report between the two task conditions. The proportion of target responses was significantly higher in the ordinal cue condition than in the shape cue condition (*t*(9) = 2.92, *p* = 0.02). Taken together, these results show that the sequential order of stimuli can be memorized reliably, with recall performance better than for color or shape, and that ordinal position is a very effective cue for retrieving other features of an item. Moreover, we found a clear recency effect across all conditions, and evidence that failure in retrieving the correct bindings between features contributed significantly to recall errors.

#### Error correlations and model fits

While the preceding analyses indicate that the ordinal positions of colors and shapes in a sequence can be memorized reliably, they do not provide positive evidence that ordinal position mediates the binding between other features. One way to discriminate between different binding mechanisms is to consider dependencies in response errors for different features. To evaluate these dependencies, we determined the proportions of response types for the first response (color) in both task conditions separately for trials in which the second response was correct (*second-target*) and trials with a swap error in the second response (*second-swap*).

We compared these response patterns to predictions from two models: The *temporal binding model* assumes that color and shape are only bound to each other indirectly via an item’s ordinal position, while the *object-based model* assumes that color and shape are bound to each directly, and the conjunction is then bound to an ordinal position. We fitted both models to each participant’s response distributions (frequencies of selecting each response option for each ordinal position of the cued item), constrained by the predicted error correlations imposed by the assumed binding mechanism in each model.

For both task conditions, in second-target trials participants tended to also select the target feature in their first response (Figure 3b and e). This is qualitatively consistent with the predictions of both models, although the object-based model tended to underestimate the proportion of target responses. The more informative results for evaluating the two models come from the second-swap trials. In the ordinal-cue condition, participants for the most part still selected the correct color in the first response when they made a swap error for shape in the second response (Figure 3c). This closely matches the prediction of the temporal binding model, in which the two responses are generated independently based on the ordinal cue. The small decrease in the proportion of color target responses in second-swap compared to second-target trials that is visible in both the data and the predictions of the temporal binding model is a result of the overall recency effect. The second-swap trials comprise more trials in which the earlier items in the sequence were cued, and for these the performance in the color report was also slightly worse.

The object-based model fails to capture the pattern of results in the second-swap trials. In this model, swap errors should arise primarily when the wrong bound object is retrieved for the cued ordinal position, so swap errors for shape should be accompanied by matching swap errors in color. This is not supported by the data.

In the shape cue condition, we find the opposite pattern in the behavioral data. In second-swap trials, participants rarely reported the target color, but rather made swap errors in their color report as well (Figure 3f). More specifically, the color selected in the first response tended to match the incorrect ordinal position selected in the second response (congruent swap errors). This is again closely matched by the temporal binding model. In this model, the color associated with the shape cue can only be retrieved indirectly via the item’s ordinal position, so an error in the retrieved ordinal position will necessarily lead to a corresponding error in the color response. The object-based model predicts that the correct color can still be retrieved reliably when the ordinal position response is wrong (since it assumes that color is directly bound to shape), which is not consistent with the behavioral results.

We also performed a formal model comparison between the two models. Since both models have the same number of free parameters, we can use the log-likelihood as a measure of the quality of fit. Consistent with the qualitative results, we found that the temporal binding model provides a better fit for the data of each of the ten participants, with a large difference in mean log likelihood, ΔLL = 75.2 ± 39.3 (mean ± SD).

### Results of Experiment 1b

In Experiment 1b, the sample stimuli were identical to Experiment 1a, but the roles of color and shape were swapped in the response phase. Participants were either cued with an ordinal position and had to select first the shape, then the color of the cued item; or they were cued with the color of one sample item and had to report the item’s shape, then its ordinal position. Additionally, participants now performed an articulatory suppression task during the sample and delay period to rule out verbal encoding strategies.

**Figure 4:**
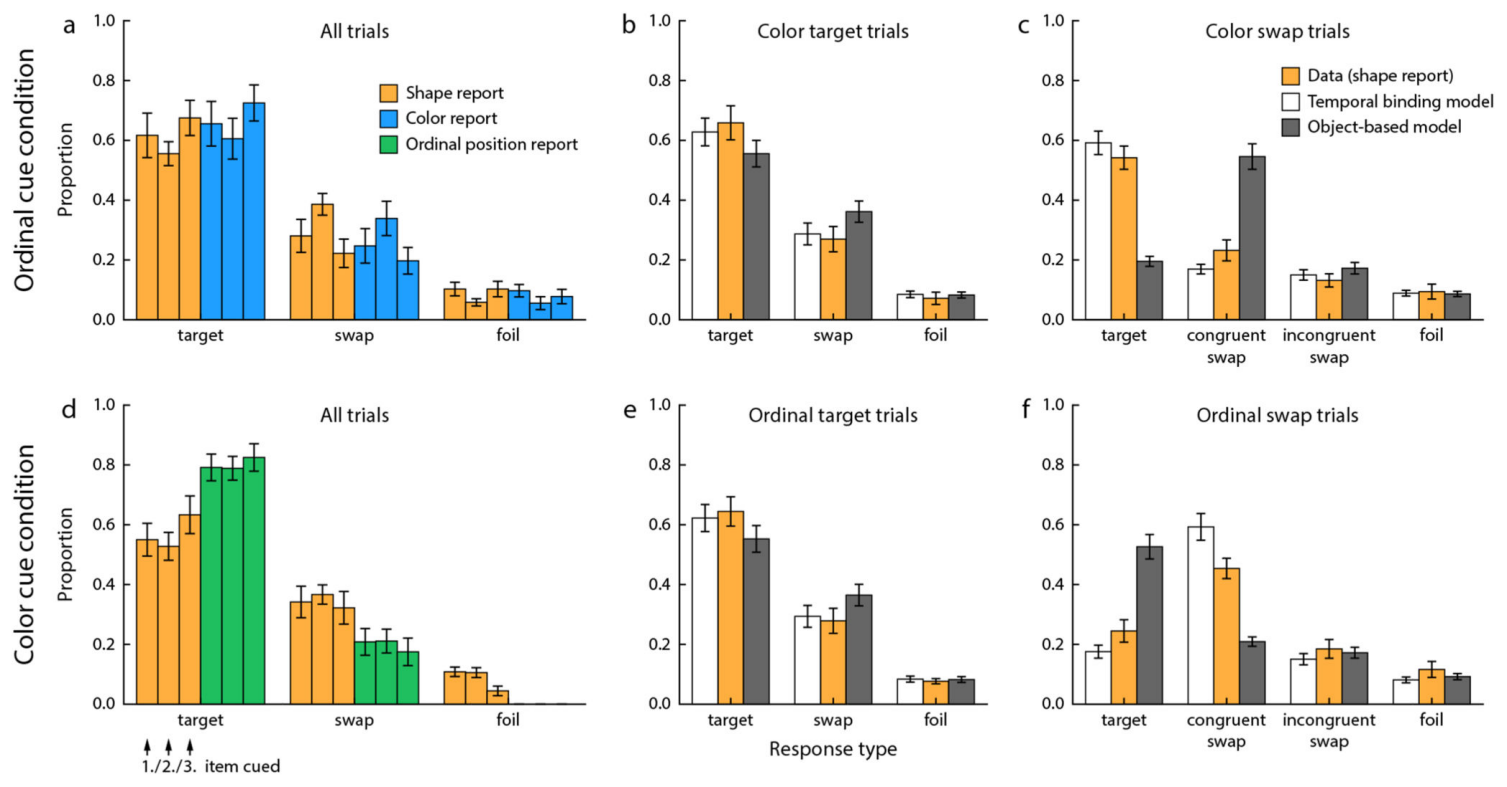
Response distributions in Experiment 1b, shown in the same format as in Figure 3.

Recall performance in the two conditions of this task is shown in Figure 4. Applying a two-way repeated measures ANOVA to the proportion of target responses in the ordinal cue condition, we found no significant effect of report feature (shape or color; *F*(1, 54) = 0.45, *p* = 0.52), no significant effect of the target item’s ordinal position (*F*(2, 54) = 3.03, *p* = 0.073), and no significant interaction (*F*(2, 54) = 0.020, *p* = 0.98). In combination with the findings from Experiment 1a, this indicates that the color report component was overall easier than the shape report, but performance was reduced for the feature reported second. Comparison of the proportions of swap and foil responses showed that misbindings contributed significantly to response errors both for shape (*t*(9) = 3.48, *p* = 0.007) and color (*t*(9) = 2.93, *p* = 0.017).

In the color cue condition, the proportion of target responses was significantly higher in the ordinal position report than the shape report (*F*(1, 54) = 26.3, *p* < 0.001). There was no significant effect of the target item’s ordinal position (*F*(2, 54) = 1.32, *p* = 0.29), and no interaction (*F*(2, 54) = 1.27, *p* = 0.30). Misbindings again contributed significantly to response errors for shape (*t*(9) = 6.78, *p* < 0.001). The difference in proportion of target responses for shape between the two task conditions did not reach significance (*t*(9) = 2.04, *p* = 0.07), suggesting that color and ordinal position have similar efficacy when used as cue.

Figure 4b-c and e-f shows the proportions of different response types in the first (shape) report for second-target and second-swap trials. The pattern qualitatively matches that observed in Experiment 1a, although recall performance was overall lower. When participants made a swap error in the color report of the ordinal cue condition, their shape report was still mostly accurate. In contrast, a swap error in the ordinal position report of the color cue condition was typically accompanied by a congruent swap error in the shape report. In both cases, the behavioral data matched the predictions of the temporal binding model, but not those of the object-based model. The formal model comparison likewise shows that the temporal binding model provided a better fit to the data of every participant (ΔLL = 19.4 ± 14.2).

### Discussion

In two tasks, we found strong support for the hypothesis that ordinal position can mediate binding between visual features, in the same manner as has previously been described for spatial location. Recall errors for different features, namely color and shape, occurred independently when an item was cued by its ordinal position (analogous to results of Bays et al., 2011; Fougnie & Alvarez, 2011), whereas congruent swap errors between the reported ordinal position and other features occurred when an item was cued by shape or color (analogous to results of Schneegans & Bays, 2017). Results closely matched the predictions of a model implementing binding via ordinal position, while the predictions of an object-based binding model clearly diverged from the behavioral data.

We note that the object-based model implemented here is just one possible realization of the concept of bound object representations, chosen primarily to be symmetrical to the temporal binding model. Other realizations of this idea might yield somewhat better fits to the data, but we believe the qualitative results, especially in the ordinal cue condition, provide robust evidence against this class of models. If color and shape of an object were strongly bound to each other in working memory, we would expect whole objects to be swapped when ordinal position cannot be retrieved reliably, but we found no evidence of this.

We found evidence for indirect binding via the ordinal position despite the fact that ordinal position always had to be reported *after* the color or shape report, so there was no incentive in the experimental procedure to retrieve an item’s ordinal position first. Furthermore, the special role of ordinal position cannot be explained simply by a higher precision of ordinal position memory compared to memory for other visual features. In Experiment 1b, ordinal position cues and color cues showed similar effectiveness for the recall of shapes, but we still observed nearly opposite patterns of error correlations between the two conditions.

There are additional sources of errors that are not accounted for in either model considered here, namely item similarity in color or shape. Participants may retrieve the features of a non-target item whose cue feature is similar to the given cue, or may select an incorrect response option because it is similar to the feature retrieved from memory. We used a large minimum feature distance between color and shape values of all items within a trial to reduce the frequency of such errors, and an analysis of feature similarity effects showed that their impact was relatively small (see Appendix A). Critically, such similarity effects cannot explain the specific error correlations observed in the behavioral data, and if they were a dominant cause of errors in this task, neither model should fit the data well.

## Experiment 2

Having found evidence that ordinal position can take the same role in mediating feature binding as spatial location, we next aimed to investigate the relationship between these two. Is either temporal order or location dominant in visual working memory (Delogu et al., 2012; Rondina, Curtiss, Meltzer, Barense, & Ryan, 2017), or can we perhaps switch freely between different binding mechanisms depending on task demands?

We employed a new dual-report task in which participants viewed colored disks that were presented sequentially at different locations. Participants then received either an ordinal or a spatial cue, and had to report the color of the cued item as well as the remaining feature (ordinal position or spatial location). Similar to Experiment 1, we analyzed error correlations between the two responses to determine whether an item’s color is retrieved directly based on the given cue, or indirectly via the second reported feature.

We made some modifications to the task design in order to adjust memory demands. Our approach relies on the occurrence of misbinding errors between an item’s ordinal position and its spatial location, and in pilot experiments we found memory for sequences of locations to be very reliable. We increased the number of sample stimuli to five, each of which could appear at one of eight locations around the fixation point. To limit the difficulty of color recall with this higher set size, we used a fixed set of highly distinguishable colors, and we fixed the color of the last item in each trial to be white. To discourage verbal encoding of colors, we employed the same articulatory suppression task as in Experiment 1b.[Fig F5]

### Methods

#### Participants

Ten new participants (4 male, 5 female, 1 non-binary, age 24.2 ± 4.2 years) performed Experiment 2 after giving informed consent. All participants reported normal or corrected-to-normal visual acuity and showed normal color vision. The experiment was completed in a single session of one to one and a half hours.

#### Procedure and stimuli

The apparatus was the same as in Experiment 1. Participants initiated each trial with a mouse click, and were presented with a white fixation point (diameter 0.25 dva) at the center of a medium-grey screen. Once stable fixation was detected, the articulatory suppression sample consisting of three random letters was shown above the center of the screen for 1000 ms, followed by a 1000 ms blank interval.

Next, a sequence of five colored disks with a diameter of 1.5 dva was shown, each for 500 ms with a 500 ms inter-stimulus interval. The colors of the first four disks were drawn randomly without repetition from a fixed set of five colors taken from a color wheel in CIELAB space with a luminance of 60, centered at position [10, 10] in the *ab*-plane and with a radius of 50. Colors were equally spaced on this color wheel, starting with a color angle of zero. The fifth disk was always white, and participants were informed about this beforehand. Each disk’s location was drawn randomly without repetition from a fixed set of eight possible locations on the corners and edge-midpoints of a square with an edge length of 6 dva, centered on the fixation point.

After a 1000 ms retention interval following the presentation of the last sample stimulus, a response cue was presented. In the *ordinal cue condition*, this was one of the digits from 1 to 5 presented at the center of the screen. After at least 500 ms, once the mouse was moved by the participant, the response options appeared. The six color response options (the set of five hues plus white) were displayed horizontally aligned above the sample stimulus area, in randomized order. The eight possible stimulus locations were shown as white outlines. Participants first had to click on one of the colors, then on one of the locations to make their response.

In the *spatial cue condition*, the cue was a white outline at the location of one sample stimulus. The color response options were shown as in the ordinal cue condition, and response options for the ordinal position were displayed as white circles containing the digits 1 to 5 below the sample display area. Participants had to first click on one of the colors, then on one of the ordinal position options.

Participants completed three consecutive blocks of 40 trials in each task condition (120 trials per condition in total; due to a technical problem, one participant completed only 112 trials in the ordinal cue condition). The order of conditions was counterbalanced across participants, and each block contained eight trials for each of the five possible ordinal target positions.

#### Response analysis

We performed the same statistical analyses on the behavioral results as in Experiment 1. Additionally, we analyzed the effects of temporal and spatial distance between sample items on the probability of swap errors in the color report. We measured spatial distance in discrete steps around the square of possible stimulus locations, and temporal distance as the absolute difference in ordinal position. For each combination of spatial and temporal distance, we determined the selection probability as the ratio of instances in which the color of a non-target item with that distance to the target was selected, to the total number of non-target items that had that distance to the target across all trials.

We also employed the same type of model fits. The temporal binding model is defined as before, except that the two features bound to the ordinal position of an item are now color and location. For the ordinal cue condition, the model predicts independent report errors, such that

(9) $$\mathrm{Pr}\left(m_{\text{col}},m_{\text{loc}}|m_{\text{ord}}\right)=\mathrm{Pr}\left(m_{\text{col}}|m_{\text{ord}}\right)\mathrm{Pr}\left(m_{\text{loc}}|m_{\text{ord}}\right).$$

Here, *m*_col_ and *m*_loc_ are the ordinal indices of selected response option in the color and location response, respectively, and *m*_ord_ is the ordinal cue value. These can take values of 1 to 5 for features that were present in the trial, and 6 for the foil options in color and location report (for location, the three foil options in each trial are collapsed). For the spatial cue condition, the model predicts that the color of the cued item is retrieved indirectly via its ordinal position, yielding

(10) $$\mathrm{Pr}\left(m_{\text{col }},m_{\text{ord }}|m_{\text{loc }}\right)=\mathrm{Pr}\left(m_{\text{col }}|m_{\text{ord }}\right)\mathrm{Pr}\left(m_{\text{ord }}|m_{\text{loc }}\right).$$

As an alternative we fit the data with a *spatial binding model*, in which color and ordinal position are bound independently to an item’s spatial location (consistent with the model of Schneegans & Bays, 2017). This model predicts that the color response in the ordinal cue condition is retrieved indirectly via the selected location,

(11) $$\mathrm{Pr}\left(m_{\text{col }},m_{\text{loc }}|m_{\text{ord }}\right)=\mathrm{Pr}\left(m_{\text{col }}|m_{\text{loc }}\right)\mathrm{Pr}\left(m_{\text{loc }}|m_{\text{ord }}\right).$$

In the spatial cue condition, the model predicts independent response errors for color and ordinal position:

(12) $$\mathrm{Pr}\left(m_{\text{col }},m_{\text{ord }}|m_{\text{loc }}\right)=\mathrm{Pr}\left(m_{\text{col }}|m_{\text{loc }}\right)\mathrm{Pr}\left(m_{\text{ord }}|m_{\text{loc }}\right).$$

The conditional probabilities for each feature pair were obtained directly from the observed response frequencies as described for Experiment 1.

#### Gaze analysis

We analyzed the eye tracking data from the response phase of the ordinal cue condition to detect signatures of automatic location recall. We smoothed the raw eye-tracking data with a Butterworth filter, and segmented the data into a sequence of saccades (minimum amplitude 0.1 dva, minimum peak velocity 25 dva/s) and fixations (minimum duration 50 ms). Manual inspection confirmed that this yielded a reasonable segmentation of the eye tracking data.

Participants had to keep their gaze within 2 dva of the central fixation point during the stimulus presentation and delay period (failure to do so was detected online and resulted in abortion of the trial). We determined the direction of the first saccade following cue onset in each trial. Saccades were included in the analysis if they occurred within 1000 ms of cue onset, and if their landing point was within a square with an edge length of 7.5 dva around the screen center (extending to the outer edges of the location response fields). Saccades landing outside this region were typically directed towards the area of the color response fields, and thus not informative regarding location recall.

We classified the directions of these saccades into eight bins of equal width, centered on the directions of the eight location response fields. We then coded the saccade direction bin in each trial relative to the location of the target item (in discrete steps). Since the target location was chosen randomly in each trial, any fixed biases in saccade direction (e.g. towards the color response fields) would not lead to systematic biases in relative saccade direction.

We determined whether saccades were more likely to be directed towards the target location than would be expected by chance. The probability that a certain number *n* of saccades falls into any of the eight bins assuming a uniform distribution of saccade directions is given by a binomial distribution, Binom(*n, N*, 1/8), where *N* is the total number of saccades included in the analysis. We compared for each participant whether the number of saccades towards the target location was greater than would occur by chance in 95% of cases. We further tested whether across participants, the number of saccades towards the target location exceeded the number of saccades in every other direction bin relative to the target, using paired-sample t-tests.

### Results

#### Recall performance

**Figure 6:**
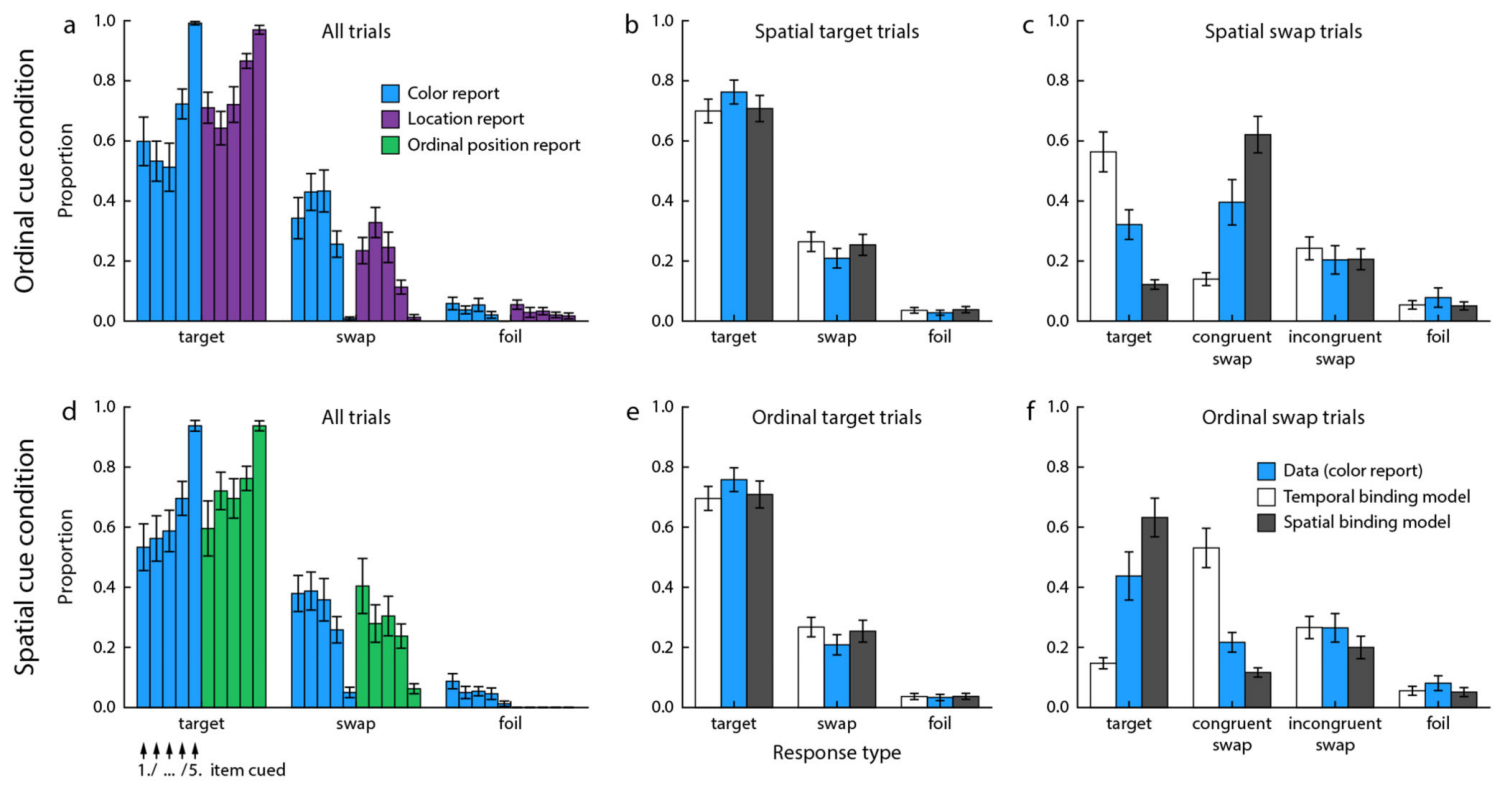
Response distributions in Experiment 2, shown in the same format as in Figure 3. In panels a and d, results are shown separately for the five possible ordinal positions of the target item.

We measured recall performance when participants had to report the color and location of an item indicated by its ordinal position (ordinal cue condition), and when they had to report color and ordinal position cued with the item’s location (spatial cue condition). In the ordinal cue condition, the proportion of correct responses was significantly higher for the location report than the color report (Figure 6a; two-way repeated measures ANOVA, *F*(1, 90) = 29.8, *p* < 0.001). This proportion was also significantly modulated by the ordinal position of the target item (*F*(4, 90) = 24.7, *p <* 0.001), with both a primacy and a recency effect visible in the data. There was also a significant interaction between these factors (*F*(4, 90) = 5.11, *p* = 0.0023), which can be attributed to the fact that color report performance was particular high in the trivial case that the last item in the sequence was cued (which was always white). The proportion of swap errors compared to that of foil errors was significantly greater than would be expected if all errors were random guesses, both for the color report (*t*(9) = 3.76, *p* = 0.004) and the location report (*t*(9) = 5.32, *p* = 0.005).

In the spatial cue condition (Figure 6d), we found a significantly higher proportion of target responses in the ordinal position report than in the color report (*F*(1, 90) = 6.806, *p* = 0.028); however, this effect was no longer significant when we excluded foil trials from the color report for a fairer comparison (*F*(1, 90) = 3.63, *p* = 0.089). There was a significant effect of the target item’s ordinal position (*F*(4, 90) = 14.8, *p <* 0.001), and no interaction between these factors (*F*(4, 90) = 1.42, *p* = 0.25). The proportion of swap errors compared to foil errors was not significantly greater than expected by chance for the color report in this condition (*t*(9) = 0.59, *p* = 0.57). Comparing the proportion of target responses for the color report between the two task conditions, we did not find a significant difference (*t*(9) = 0.44, *p* = 0.67), indicating that ordinal and spatial cues are similar in their effectiveness for retrieving a memorized color.

**Figure 7:**
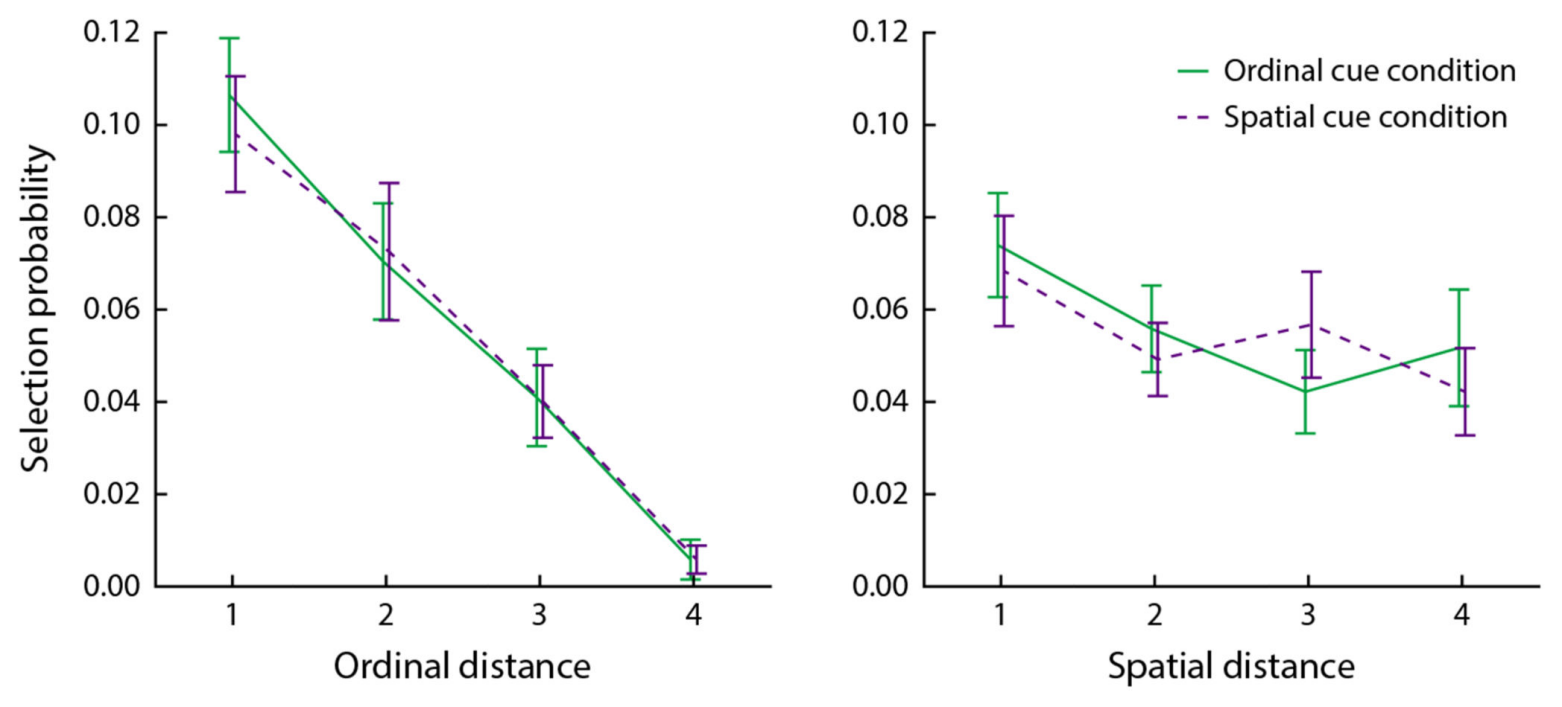
Effects of temporal and spatial distance on selection probability for non-target colors. Plots show the probability that any non-target response option is selected in the color report, as a function of the temporal distance (absolute difference in ordinal position) or spatial distance (number of discrete steps over possible stimulus locations) between the selected non-target and the target item.

We further evaluated the effects of spatial and temporal proximity on color response errors in the two task conditions. We determined the probability that any non-target color was selected dependent on how close it was to the cued target item in the memory sample presentation, both in time (absolute difference in ordinal position) and in space (in discrete steps along the eight possible stimulus locations; Figure 7). In both task conditions, we found that the probability of selecting the color of a non-target item was significantly modulated by the non-target’s ordinal distance to the target (ordinal cue condition: *F*(3, 144) = 26.6, *p* < 0.001; spatial cue condition: *F*(3, 144) = 26.7, *p* < 0.001), but not its spatial distance (*F*(3, 144) = 2.39, *p* = 0.091 and *F*(3, 144) = 2.40, *p* = 0.090), with no significant interaction (*F*(9, 144) = 1.17, *p* = 0.33 and *F*(9, 144) = 0.85, *p* = 0.57).

#### Error correlations and model fits

We considered two possible binding mechanism to explain performance in this task: *Temporal binding*, in which an item’s color and location are directly bound to its ordinal position in the sequence, and only indirectly to each other, and *spatial binding*, where color and ordinal position are directly bound only to an item’s location. To discriminate between them, we again turn to the dependencies of response errors across the two responses in each trial.

Figure 6b-c shows the proportions of target, swap, and foil responses in the color report of the ordinal cue condition, separately for trials with a correct spatial response and for spatial swap trials. Predictions of ordinal and spatial binding model are shown for comparison. While model predictions for trials with correct spatial response are similar to each other and both match the behavioral data, the models make clearly divergent predictions for spatial swap trials: In the temporal binding model, most color responses should still be correct, since an item’s color can be retrieved directly from the ordinal cue; in the spatial binding model, a swap error in the spatial response should typically be accompanied by a corresponding swap error for color. In the behavioral data, we found a pattern that is intermediate between these predictions. There was a substantial proportion of swap errors in which the color corresponding to the incorrectly chosen location was selected, but also a substantial proportion of correct color reports despite the occurrence of a spatial swap.

The behavioral results and model predictions for the shape cue condition are shown in Figure 3e-f. Here, the predictions of the two models are reversed: The temporal binding model predicts correlated swap errors between color and ordinal position, while the spatial binding model predicts that swap errors should occur independently in the two reports. Again, the observed behavioral results were intermediate between the two model predictions. There was a relatively large proportion of correct responses in ordinal swap trials, but still a higher number of congruent swap errors than predicted by the spatial binding model.

A formal comparison of the two models’ quality of fit likewise produced ambiguous results. For seven of the ten participants, the temporal binding model provided a better fit, but the mean difference in log likelihood was small (Δ*LL* = 1.47 ± 20.7) and in favor of the spatial binding model. To elucidate the cause of these ambiguous results, we also fitted each participant’s data with mixtures of the spatial and temporal binding models and determined the optimal mixture weights (see Appendix B). We found both a large variability across participants and ambiguity within participants, reflected by similar weights for the two models.

We also considered the possibility that participants may be able to flexibly switch the binding mechanism to best match the task demands, such that for each task condition the two report features would be bound directly to the cue feature. However, in each task condition, the direct binding model (temporal binding for ordinal cue condition, spatial binding for spatial cue condition) performed better than the alternative with indirect binding in only about half of the participants (ordinal cue: 5 out of 10, Δ*LL* = 1.52 ± 10.4 in favor of spatial binding; spatial cue: 6 out of 10, Δ*LL* = 1.83 ± 9.27 in favor of spatial binding).

#### Eye movements

**Figure 8:**
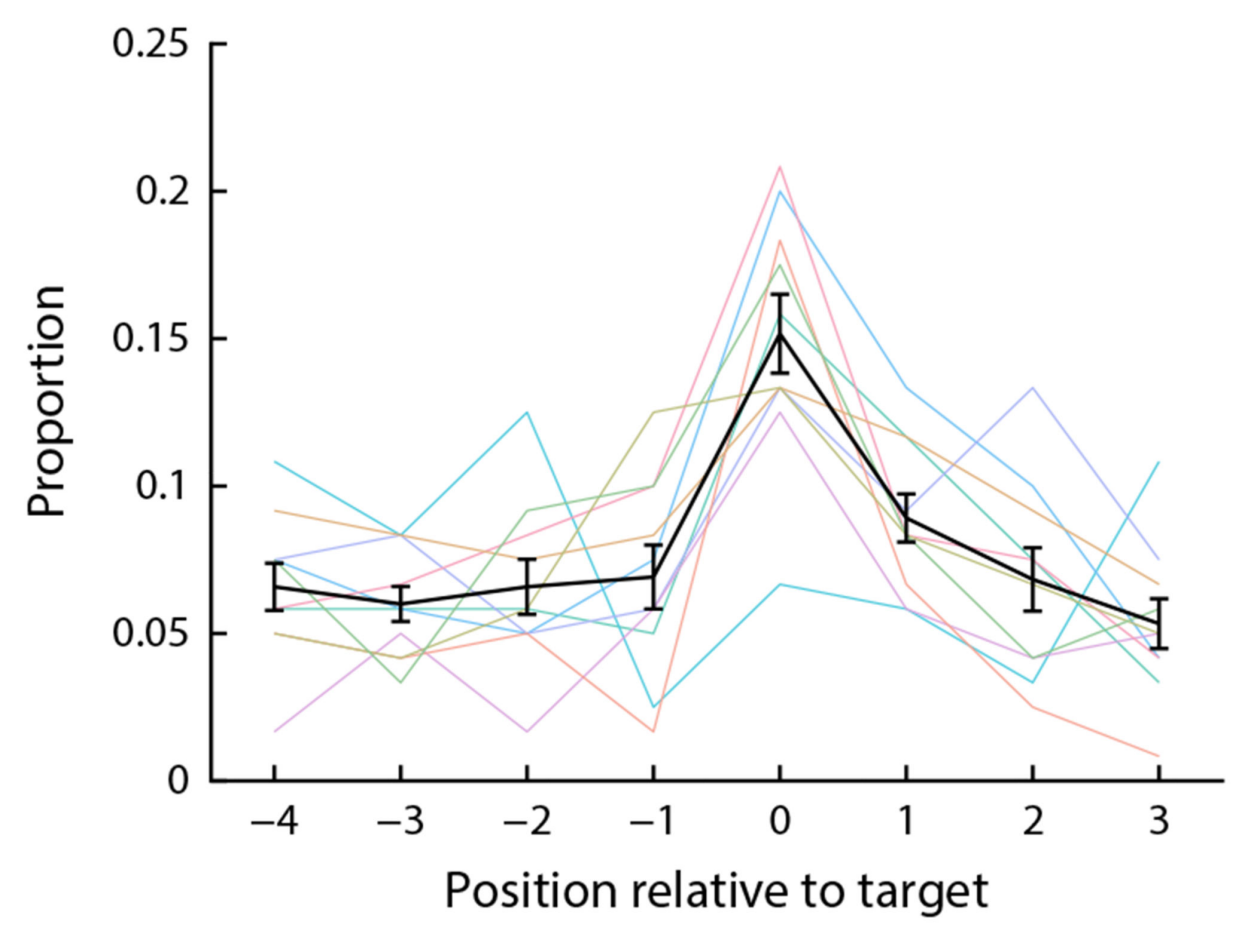
Direction of first saccade after cue onset in the ordinal cue condition, aligned to the direction in which the target item was located in each trial.

As an additional approach to elucidate the mechanism for retrieving the cued item in this task, we analyzed eye movements in the ordinal cue condition. If the location of the item indicated by the ordinal cue is retrieved automatically during cue processing, we expected to see a bias in gaze direction towards the cued location. This is indeed what we found (Figure 8): The first saccade after presentation of the ordinal cue (always occurring within 1 second of the cue, and before a color response was made) was directed more frequently towards the target location than towards any of the other sample locations (paired t-tests, all *t*(9) ≥ 4.68, *p* ≤ 0.0012). For seven of the ten participants, the proportion of saccades towards the target location was greater than would be expected by chance (5% significance level).

### Discussion

In this experiment, we observed that both the locations and the ordinal positions of memorized items could be recalled reliably, and ordinal and spatial cues showed similar effectiveness for retrieving an item’s color. We further found that recall errors in the color report were driven primarily by swaps between items that were presented close to each other in time, while spatial proximity had a comparatively small effect on swap probability. This is consistent with previous results (Sapkota, Pardhan, & van der Linde, 2016; Schneegans et al., 2021). We note that this does not imply that ordinal position is the dominant feature for mediating binding, but merely indicates that adjacent ordinal positions were more confusable than adjacent locations in this task. It is likely that this is in part due to the general effects of the target’s ordinal position on recall performance—namely, that observers are more likely to confuse features of two items if they are both remembered relatively poorly, such as when they occurred in intermediate ordinal positions in the sequence of sample stimuli. In contrast, accuracy did not vary strongly with spatial location.

To determine the binding mechanism employed in this task, we analyzed the error correlations between the two responses in each task condition. We found that in both task conditions, the results were intermediate between predictions based on either a purely spatial binding model or a purely temporal binding model. Specifically, in trials with a swap error in the second report, we found both a high proportion of correct responses and of congruent swap errors in the color report, while each model predicts a clear dominance of one of these in different task conditions. This finding does not seem to be due to a general failure of the models to capture the true sources of errors, since the proportions of both incongruent swap errors and foil responses for color were low and qualitatively matched the model predictions.

The behavioral results are also inconsistent with an entirely flexible binding mechanism, in which either ordinal position or location can be used to mediate binding depending on task demands. Under such a mechanism, we would expect participants to preferentially bind the two features to be reported directly to the cue feature in each task condition, but we did not find evidence for such a pattern. This suggests that a mixture of spatial and temporal binding is employed, with contributions of both mechanism within individual participants as well as variability of their weighting across participants. Such variability is consistent with recent findings on incidental encoding of task-irrelevant spatial and temporal structure in change detection tasks (Heuer & Rolfs, 2021), but fully quantifying it is beyond the scope of the present study.

Finally, the analysis of eye movements in the ordinal cue condition revealed a gaze bias towards the location of the cued sample item, similar to what has previously been reported in working memory studies even when location was entirely irrelevant for the task (van Ede, Chekroud, & Nobre, 2019). The results indicate that location information was often retrieved in the earliest interval of the report phase, at a time before the color response was made. This suggests some automatic retrieval of location information in response to the ordinal cue. Unfortunately, the gaze measure only yields a lower limit for how often this rapid retrieval of spatial information occurs (since the absence of an eye movement does not demonstrate that the location was not retrieved), and it cannot be readily applied to the spatial cue condition.

## General discussion

Our visual perception is structured by time and space, and both the spatial layout and the temporal order in which we perceive individual stimuli are critical to extract meaning from the visual input. In two experiments, we examined the role of presentation time for the binding between visual features, and its relationship to the role of space. To elucidate the binding mechanism, we compared the observed patterns of error correlations to predictions from three theoretical models, namely spatial binding, temporal binding, and an object-based model.

In Experiment 1, we found strong and consistent evidence that object shape and color are bound directly to the ordinal position at which an item appeared, and are bound to each other only indirectly via this ordinal position, consistent with the temporal binding model. When an item was cued by its ordinal position, report errors for shape and color were nearly perfectly independent of each other, analogous to previous findings for cueing an item by its location in a simultaneously presented array (Bays et al., 2011; Fougnie & Alvarez, 2011). When the target was cued by its color or shape, a swap error in reporting its ordinal position was typically accompanied by a congruent swap error in reporting the other feature. This likewise mirrors previous findings supporting the spatial binding model (Kovacs & Harris, 2019; Schneegans & Bays, 2017). This indicates that for sequentially presented items, time or ordinal position can take the same role in mediating binding of other features as has previously been proposed for spatial location.

We note that these results do not contradict earlier findings that there is a general cost to presenting items sequentially rather than simultaneously (Allen et al., 2006; Gorgoraptis et al., 2011). We used a set size of only three items in Experiment 1, compared to five or six items in comparable studies with simultaneous sample presentation (Bays et al., 2011; Fougnie & Alvarez, 2011), and still observed a significant proportion of swap errors in all reports.

The finding of independent report errors in the ordinal cue condition is in conflict with object-based accounts of feature binding, which assume that an item’s visual features are memorized in a closely bound form(Luck & Vogel, 1997; Treisman & Zhang, 2006). These would predict that whole bound objects should be swapped in this condition, rather than individual features. In combination with previous results, this finding indicates that object-based representations do not materially contribute to feature binding in visual working memory in a wide range of experimental tasks.

There is some evidence that in long-term memory, binding of features within an object is more robust than binding of objects to spatiotemporal context or to other objects (Mayes, Montaldi, & Migo, 2007). It is conceivable that similar patterns would also be observed in visual working memory for naturalistic stimuli (which likely relies on long-term memory for object categories), compared to the arbitrary combinations of abstract features used in the present study. However, one working memory study testing binding between objects and colors with naturalistic stimuli still observed only indirect binding via space (Kovacs & Harris, 2019).

In Experiment 2, we aimed to contrast the spatial and temporal binding models to determine whether one form of binding was dominant. For instance, it may be that temporal binding is indeed the primary binding mechanism in visual working memory, and location is treated like other features such as color. If the binding of locations to ordinal positions is very reliable, such a mechanism may still appear like spatial binding in a task where ordinal position memory is not tested. The task design with sample stimuli separated both in time and space, and both ordinal position and location being task relevant, allowed us to test this possibility.

The results in this experiment were more ambiguous, but nonetheless informative.We found evidence for both location and ordinal position mediating binding, with comparable contributions from each to error correlations in both task conditions. This speaks against an overall dominance of either mechanism. It does not support the idea that binding is always mediated by space, and objects are internally remapped when they are presented at the same location (Abrahamse et al., 2014). Since stimuli in this experiment always appeared at different locations, no internal remapping would have been necessary, and we should have seen only signatures of spatial binding.

Likewise, the results do not support the view that temporal binding is the primary mechanism, with sequential attention imposing a temporal order even when stimuli are presented simultaneously (Swan & Wyble, 2014). If this were the case, we should have seen only signatures of temporal binding in this experiment. Finally, we also did not find evidence that participants strategically switched between different binding mechanisms, such that they would always bind the reported features directly to the cue feature.

We propose that the results support a working memory representation with a mixed code, in which conjunctive population codes for binding between surface features (like color, shape, or orientation) and location, between surface features and ordinal position, and between location and ordinal position coexist. The binding of locations and ordinal positions appears to be the most robust, reflected in high recall performance for either of these when cued with the other one, and in the evidence from the gaze analysis for rapid automatic retrieval of location when cued with ordinal position. The ability to accurately represent sequences of locations is likely important for representing movement trajectories in perception and motor planning (e.g. Hayhoe, Shrivastava, Mruczek, & Pelz, 2003).

If the location associated with a cued ordinal position (or vice versa) is retrieved first, then either of these may be used to retrieve an item’s color; and if an error occurs in retrieving the former, then two incongruent cues would compete with each other in the retrieval of the color, leading either to selection of the correct target color or a congruent swap error.

The form of mixed coding proposed here based on behavioral results is compatible with several recent studies investigating neural activity patterns during memory-guided saccade tasks in macaque monkeys (Murray et al., 2017; Parthasarathy et al., 2019; Spaak, Watanabe, Funahashi, & Stokes, 2017). These studies consistently found a mixture of stable (time-invariant) coding of a saccade target location and dynamic activity patterns that allowed decoding the time since stimulus presentation. Another study decoded task variables from delay-period EEG activity in human participants performing a delayed reproduction task for orientation stimuli (Wolff, Jochim, Akyürek, Buschman, & Stokes, 2020). They likewise found both stable subspaces in the neural code that allowed decoding of memorized orientations independent of time (but specific to stimulus location), and temporal dynamics in the memory representation that allowed decoding timing information.

Moreover, Cueva et al. (2020) recorded neural activity in monkeys performing different short-term memory tasks, and they observed that the precision with which they could decode stimulus timing varied depending on whether this timing was relevant in the context of the current task. This finding supports the notion that the decoded temporal information is indeed utilized by the neural system, and not merely a signature of passive memory decay. One caveat in the interpretation of the neural decoding results in the context of the present study is that none of the experiments attempted to decode separate timing information for multiple stimuli, so it is not clear whether different presentation times for individual stimuli are reflected in these temporal dynamics.

One possible confounding factor in the present experiments is that the separate and sequential responses for the two reported features in each trial might bias results towards more independent recall errors. This was suggested by a recent study that employed a combined response display for simultaneous color and orientation reports (Sone, Kang, Li, Tsubomi, & Fukuda, 2021), and found significant correlations between response errors when using location cues, contrary to previous results (Bays et al., 2011; Fougnie & Alvarez, 2011). We note that the study also found similar, albeit weaker, error correlations in a task with sequential reports, suggesting that other factors in the experimental design contributed to this outcome. Importantly, the mode of response cannot explain our results in the different cueing conditions of Experiment 1, where we observed nearly opposite patterns of error correlations despite closely matched response procedures.

Another limitation of the experimental design employed here is that it explicitly requires participants to memorize the ordinal position of each feature. We can therefore not draw strong conclusions about the role of temporal order if it is merely incidental. However, a recent study by Heuer and Rolfs (2021) found evidence that the temporal structure of a stimulus display is encoded in working memory even when it is task-irrelevant, and affects recall performance. These authors employed a change detection task in which sample stimuli were presented sequentially and at different locations. Change detection performance was impaired if either the spatial or temporal structure of the sample array was removed in the test display, while omitting other task-irrelevant features like color or size had no comparable effect.

Taken together, these complementary behavioral results and the neurophysiological findings point toward an important role for time in structuring visual working memory representations that may mirror the role of space. Similar views have been emerging in the field of long-term memory, with the proposal that time cells in the hippocampus encode temporal structure in a way that is analogous to the role of place cells (MacDonald, Lepage, Eden, & Eichenbaum, 2011; Umbach et al., 2020). Fully understanding the conributions and interactions of space and time in memory will be a challenge to be met by future research.

## Supplementary Material

Supplementary Material

## Figures and Tables

**Figure 5 F5:**
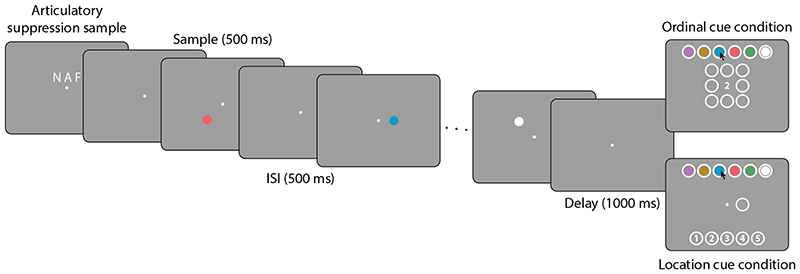
Behavioral task in Experiment 2. A total of five colored disks were presented sequentially and at different locations in each trial.
