# Supplementary material for: Role of time in binding features in visual working memory
Source: Psychol Rev. Author manuscript; Available in PMC 2023 Feb 4. (PMC7614141; doi:10.1037/rev0000331)
Supplement: Supplementary Material [file EMS141025-supplement-Supplementary_Material.pdf]

Appendix A

Effects of feature similarity in Experiment 1

Methods

Previous work has shown that swap errors in delayed reproduction tasks can often be attributed to imprecision in memory for the cue features (Bays, 2016; Emrich & Ferber, 2012; Oberauer & Lin, 2017; Rerko, Oberauer, & Lin, 2014; Schneegans & Bays, 2017), in that items that are similar to the target item in the cue feature are more likely to be erroneously selected. To investigate whether analogous mechanisms also contribute to the discrete response errors in Experiment 1, we analyzed the effect of cue feature similarity on response choice.

The response options given in each trial included the target feature, the two non-target features, and a foil feature (except for the ordinal position report, where no foil response was possible). Each of the non-target response options was associated with a certain cue feature in the sample array. We grouped the non-target response options according to how similar their associated cue feature was to the given cue in each trial (which is identical to the cue feature of the target item), and determined the probably that a non-target response option was selected separately for each group.

For the ordinal cue condition, we grouped the non-target response options according to each item's ordinal distance to the target item (in discrete steps). For the shape cue and color cue conditions, we discretized the feature distance (absolute angular difference of feature values in the circular shape and color spaces) into four bins. Bin centers were chosen such that the expected number of items falling into each bin would be equal (due to the minimum distance between the features of different items within a trial, the distributions of feature distances was not uniform). We determined the selection probability for each group as the ratio between the number of times a non-target response option from each group was selected and the number of times a non-target response option from each group was offered.

Errors in the response selection may also occur due to imprecision in memory for the report feature, which may result in selection of a (swap or foil) response option that is similar to the target's report feature. We grouped foil and swap response options based on their ordinal or feature distance to the target feature in the same way as described for cue similarity, and determined the selection probability for each group.

We assessed the influence of cue and report feature similarity on response selection using one-way repeated measures ANOVAs (in the case of ordinal position with only two possible distance values, this is equivalent to a paired samples t-test). For cue feature similarity, foil responses are not included in the analysis, but are shown in the figures for comparison (proportions of swap and foil responses in each task were compared in the main results).

Results

Results for Experiment 1a are shown in Figure A1. In the ordinal cue condition, there was a significant effect of cue similarity (i.e., ordinal proximity) on the selection of non-target response options both for the color report ($F(1) = 5.75$, $p = 0.040$) and the shape report ($F(1) = 11.7$, $p = 0.008$). Response selection in the color report also showed a significant effect of report feature similarity ($F(3) = 3.05$, $p = 0.046$), whereas we found no such effect in the shape report ($F(3) = 0.73$, $p = 0.54$). In the shape cue condition, we did not find an effect of cue similarity in the color report ($F(3) = 2.11$, $p = 0.12$), but there was a significant effect in the ordinal position report ($F(3) = 3.97$, $p = 0.018$). The effect of report feature similarity in the shape cue condition was not significant for the color report ($F(3) = 1.07$, $p = 0.38$), but it was significant for the ordinal report ($F(1) = 16.6$, $p = 0.003$).

The corresponding results for Experiment 1b are shown in Figure A2. In the ordinal cue condition, response selection was affected by cue similarity for both the shape report ($F(1) = 10.0$, $p = 0.011$) and the color report ($F(1) = 6.09$, $p = 0.036$). There was no

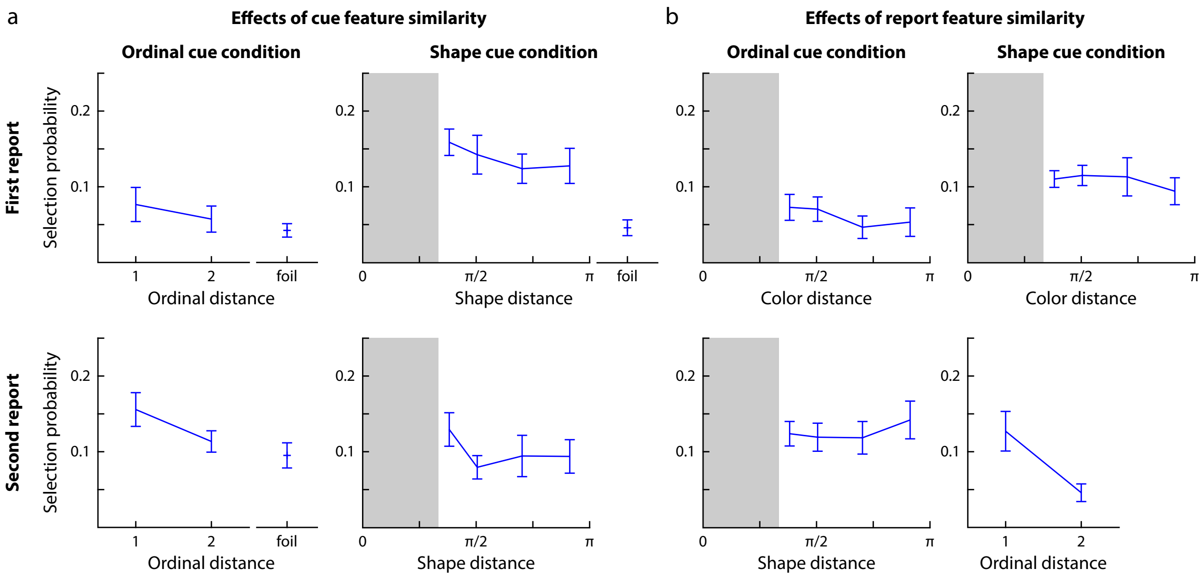

Figure A1

Effects of feature similarity on response selection for each task condition and response in Experiment 1a. The probability of selecting a certain non-target response option is shown as a function of the distance between the cue feature associated with that option and the actual cue (a), and as a function of the similarity between the report feature and the report feature of the target item (b). For the effects of cue feature similarity, the probability of selecting the foil response option (for shape and color report) is shown separately for comparison. Data points show mean value across participants, and error bars indicates ±1 SE. The gray shaded area indicates the minimum feature distance between all items in a trial.

significant effect of report feature similarity in either report (shape: $F(3) = 1.11$, $p = 0.36$; color: $F(3) = 2.24$, $p = 0.11$). In the shape cue condition, there was no effect of cue similarity in the color report ($F(3) = 0.46$, $p = 0.71$), but we did find a significant effect in the ordinal report ($F(3) = 3.46$, $p = 0.030$). Similarity in the report feature likewise did not have an effect on color report ($F(3) = 1.87$, $p = 0.16$), but there was a highly significant effect on the ordinal report ($F(1) = 36.4$, $p < 0.001$).

In summary, effects of feature similarity were predominantly observed for ordinal position (where feature similarity takes the form of temporal proximity in the sample

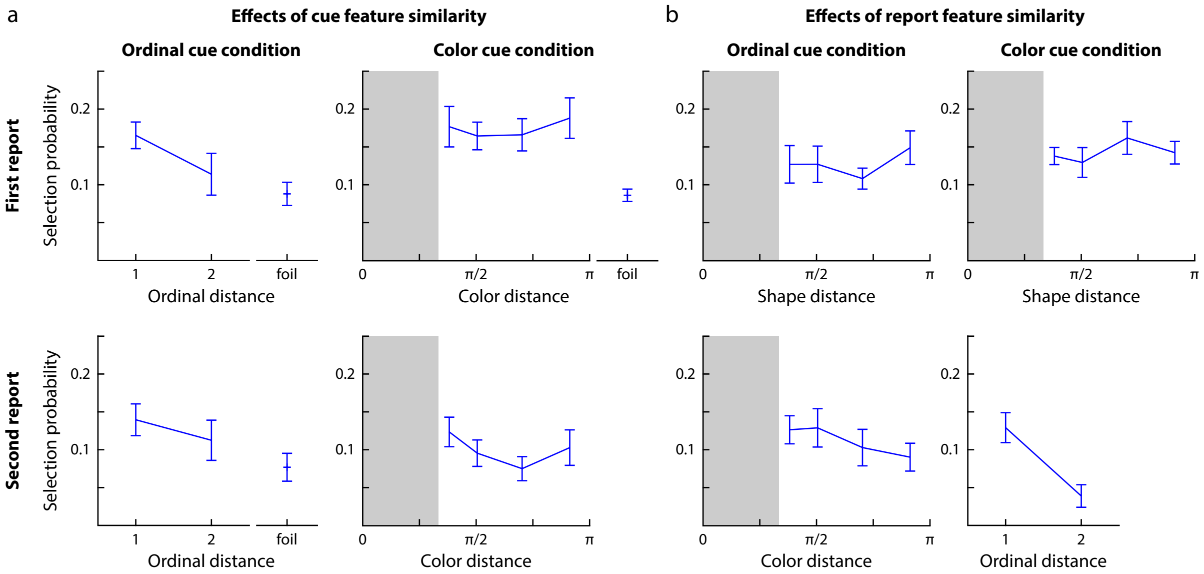

Figure A2

Effects of feature similarity on response selection for each task condition and response in Experiment 1b, shown in the same format as in Figure A1.

sequence). It should be noted that these effects may be driven by the general recency effect described in the main results, which would likely produce more swap errors between the first two items in the sequence than between the first and the third item. Effects of feature similarity in color and shape were relatively limited, indicating that the large minimum distance between feature values within a trial largely prevented confusion of items features. The finding that cue similarity had a greater effect in the second (ordinal) report than the first report for both the shape cue condition (Experiment 1a) and the color cue condition (Experiment 1b) is consistent with the assumption of indirect binding via the ordinal position.

Appendix B

Mixtures of binding models

To further quantify to what degree the predictions of the different binding models explain the behavioral results, we fit the data of each participant with mixtures of the two models considered in each experiment. For Experiment 1a and 1b, we created weight matrices $\mathbf{P}_{\mathrm{mix}}$ (with entries as described in Eq. 3 of the main manuscript) as weighted mixtures of the matrices generated for the temporal binding model and the object-based model,

$$\mathbf{P}_{\mathrm{mix}} = w\mathbf{P}_{\mathrm{tmp}} + (1 - w)\mathbf{P}_{\mathrm{obj}} \tag{13}$$

For Experiment 2, we generated mixtures of the temporal binding model and the spatial binding model in the same manner. We then determined for each participant the maximum likelihood estimate of the mixture weight w, $0 \leq w \leq 1$.

The resulting estimates are shown in Figure B1. In Experiment 1a and 1b, estimates cluster near a weight of 1 for the temporal binding model, as expected based on the model comparisons described in the main manuscript. For Experiment 2, estimated weights are broadly distributed, with some participants showing a clear preference for either temporal or spatial binding, but most showing intermediate weights.

For Experiment 2, we also tested whether the preferred binding mechanism had an effect on performance. We performed a median split of participants based on the estimated mixture weights, and compared the proportions of target responses between the two groups, separately for each task condition and for each reported feature, using independent-sample t-tests. We did not find a difference in performance in any of the comparisons (all $t(8) \leq 0.43$, $p \geq 0.68$).

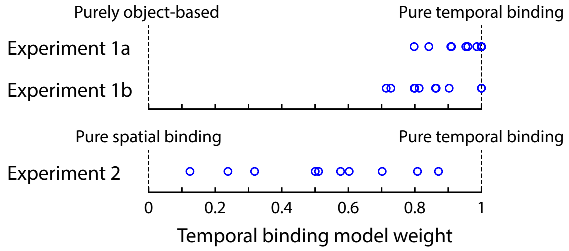

Figure B1

Maximum likelihood estimates of mixture weights for temporal binding and object based binding (Experiment 1) or spatial binding (Experiment 2).
